# Occipital condyle fracture in a patient with neck pain

**DOI:** 10.1186/1865-1380-7-5

**Published:** 2014-01-14

**Authors:** Muhammad Waseem, Ruchi Upadhyay, Husayn Al-Husayni, Samuel Agyare

**Affiliations:** 1 Lincoln Medical & Mental Health Center, 234 East 149th Street, Bronx, NY 10451, USA

**Keywords:** Craniocervical, Occipitocondyle, Occipital condyle fracture

## Abstract

**Background:**

Occipital condyle fractures (OCF) are rare traumatic injuries and are of critical clinical importance because of the anatomic considerations of the occipitoatlantoaxial joint complex. OCF can be a diagnostic challenge because of the inability to diagnose this injury with plain radiographs. This is especially true in the emergency department (ED) setting. A high degree of clinical suspicion and careful investigation of the craniocervical junction is warranted in patients presenting to the ED with head and cervical trauma.

**Findings:**

We present a case of a 45-year-old male who presented to the ED with complaints of neck pain and headache four days after an assault. The classification, clinical presentation, diagnosis, and management of his injury are discussed, and pertinent literature is reviewed.

**Conclusions:**

OCF can be easily overlooked due to multiple factors; including the conscious state of the patient or the inability to diagnose it through plain radiographs. Early recognition and diagnosis of OCF is crucial to prevent neurological involvement.

## Background

Occipital condyle fractures (OCFs) are rare traumatic injuries that can pose a diagnostic challenge, especially in the emergency department (ED) setting. They are of critical clinical importance, as is any other injury to the atlanto-occipital region, because of the anatomic considerations of the occipitoatlantoaxial joint complex. They can easily go undetected due to variable presentation and the inability to diagnose them with plain radiographs. A high degree of clinical suspicion and careful investigation of the craniocervical junction is warranted in patients presenting to ED with head and cervical trauma. We herein outline the case of a 45-year-old male who presented to the ED with complaints of neck pain and headache four days after an assault. The classification, clinical presentation, diagnosis, and management are discussed and the pertinent literature is reviewed.

## Case presentation

A 45-year-old male presented to the ED with complaints of neck pain and headache after an assault four days prior. The neck pain progressively worsened in intensity and was worse on turning his head to the right side. The patient had no fever, nausea or vomiting, dizziness or blurry vision, and denied any loss of consciousness. There was no significant past medical history.

On examination, his vital signs were as follows: temperature 36.9°C (98.4°F), heart rate 72 beats/minute, respiratory rate 20 breaths/minute and blood pressure 122/76 mmHg. He was alert and oriented. Neck examination revealed tenderness to the back of the neck. There was no swelling. Neurologic examination showed no deficits and no cervical or cranial nerve palsies. The remainder of the physical examination was unremarkable.

**Figure 1:**
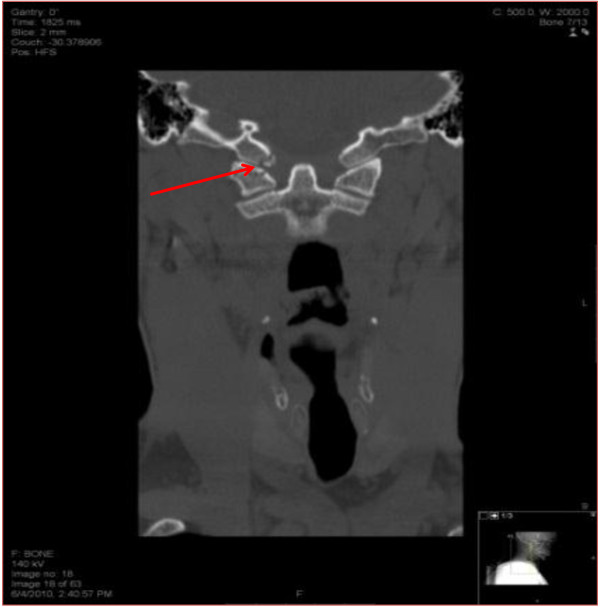
Minimally displaced fracture of the right inferior medial occipital condyle.

A CT scan of the brain was performed from the base of the skull to the vertex at a slice thickness of 2.5 mm for the posterior cranial fossa and 5 mm for the supra-tentorial compartment. The study did not reveal any gross bony abnormality. Spiral CT scan of the cervical spine was performed from the base of the skull down to the thoracic inlet at a slice thickness of 2.5 mm. A minimally displaced fracture of the right inferior medial occipital condyle was noted (Figure 1). There was a reversal of normal cervical lordosis due to muscle spasm. There was no fracture of the cervical spine and no soft tissue swelling. The cervical spine was immobilized with a cervical collar and a neurosurgical consult was obtained. The patient was managed conservatively.

## Discussion

OCFs are of critical clinical importance. A rapid diagnosis is essential to ensure the start of appropriate treatment and prevent delayed neurologic deficits. The first case of OCF was described by Sir Charles Bell in 1817 [1-8]. The diagnosis was made postmortem in a young patient who had trauma. The case was extraordinary in that, while the patient reached down to pick up something, he suddenly died due to compression by the bone fragment on the medulla [3,4]. Tuli et al. found 96 case reports of OCF between 1817 and 1994, approximately 40% of which were from postmortem studies [8]. The exact prevalence of this fracture is unknown, and the incidence, among that of severe craniocervical injuries, is reported to range from 4% to 19% [3].

Over the years, there has been an increase in the number of reported cases of OCFs. The diagnosis of OCF usually remains unrecognized as it is rarely identified on plain radiographs [2,3]. This lack of utility of plain radiographs of either skull or cervical spine, together with the variability of symptoms, poses a challenge to the diagnosis of OCF [6,9]; however, with the increasing use of CT scans, more cases are being detected [3,7,10,11]. CT scans are now considered the gold standard in diagnosing this entity [9], helping to identify any displacement or bleeding in the affected area [7].

### Classification

The first classification of OCF was proposed by Saternus in 1987, based on the type of applied strain [8,11]. The classification by Anderson and Montesano, first proposed in 1988, is now the most popular, and is based on mechanism of injury and appearance on radiographic films [6,9]; Type I is an impacted fracture, Type II is a basal skull fracture, and Type III is an avulsion fracture [6,7]. Types I and II are stable fractures and Type III is unstable [3]. Tuli et al. disagreed with Anderson and Montesano in addressing stability solely on displacement of the fracture, without considering imaging to detect injury to ligaments [8]. They proposed a new classification in 1997 and grouped Anderson and Montesano’s Type I and II as Type 1, Type III as Type 2A, and proposed a new Type 2B. Tuli’s Type 1 is a non-displaced fracture, Type 2A is a displaced fracture with intact ligaments and Type 2B is a displaced fracture with a radiographic evidence of instability of the craniocervical region.

### Clinical presentation

OCFs are diagnosed twice as often in males than in females, with mean age at presentation between 32–33 years [3]. The clinical presentation of OCF is varied and can be easily missed. Many reasons potentially contribute to the lack of identification of this fracture, such as limited knowledge of this entity, failure to diagnose it through plain radiographs, and a sub-optimal physical examination. The latter occurs because the patients are often unconscious due to associated head injury and complete examination is not possible, and in those who are conscious, neck pain may be the only presenting symptom. Neurological deficits may, however, be present in some [4]. Because of the close proximity of occipital condyles with the hypoglossal canal and jugular foramen, nerves IX, X, XI, and XII can be affected [7]. An early diagnosis of OCF is crucial to prevent development of delayed nerve palsies, even if the fracture is not initially associated with any nerve deficits. The symptoms may not be present immediately and may develop months after the trauma [5]. Injury to the hypoglossal nerve, however, may present immediately [2]. The nerve deficits can be attributed to the bone fragment pressing on nerves, or yielding of the nerve during injury or damage to the nerve [12].

### Diagnosis and management

The cranial nerve examination is essential in patients with craniocervical injury [12]. In fact, OCF should be in the differential diagnoses for patients with lower cranial injuries caused by trauma [13]. OCF is difficult to diagnose by plain film of the cervical spine. In the AP projection, an open mouth view that includes the condyles is required. It is well-known that adequate views frequently cannot be obtained at first attempt. On the lateral view, the fracture itself cannot be seen due to the overlap of multiple bones. It can, however, be suspected if there is swelling of the craniocervical pre-vertebral soft tissues, which will be obscured if the patient is intubated. A CT scan is recommended in patients with suspicion of injury to the craniocervical region in presence of enduring neck pain, even with normal plain radiographs of the cervical spine. It is also recommended if the following are noted: pre-vertebral or retropharyngeal soft-tissue swelling, loss of consciousness with substantial head injury, involvement of lower cranial nerves, and spasmodic torticollis and fractures of the upper cervical spine or occipital skull base [12,14].

A multi-detector CT scan of the cervical spine with sagittal and coronal reformatted images is the best modality in the acute setting. In the setting of trauma, a CT scan of the brain should include the lower margin of C2 unless CT of the cervical spine is also obtained. The treatment of stable OCF (Andersons and Montesano’s Type 1 and II) is conservative with a cervical collar [13]. The role of surgical therapy is controversial (noble) and may be indicated to release neurovascular compression or stabilize the craniocervical region [15].

## Conclusions

The limited knowledge of OCF, failure to diagnose it through plain radiographs, and an inability to perform a complete physical examination, are among the reasons for OCFs to be overlooked. Because of the close proximity of occipital condyles with the hypoglossal canal, jugular foramen, and nerves IX, X, XI and XII, an early diagnosis of OCF is crucial to prevent development of delayed nerve palsies. A CT scan is recommended in patients with suspicion of injury.

## Abbreviations

ED: Emergency department; OCF: Occipital condyle fracture.

## Competing interests

The authors declare that they have no competing interests.

## Authors’ contributions

MW contributed to the concept, design and revision of the manuscript for important intellectual content. RU carried out acquisition of data and drafted the manuscript. HA contributed with radiological findings and related intellectual content. SA gave final approval of the version to be published. All authors read and approved the final manuscript.
